# *catena*-Poly[[diaqua­cobalt(II)]-bis­(μ-4-fluoro­benzoato-κ^2^ *O*:*O*′)]

**DOI:** 10.1107/S1600536809014913

**Published:** 2009-04-30

**Authors:** Fu-Fu Zhou, Bi-Song Zhang

**Affiliations:** a College of Materials Science and Chemical Engineering, Jinhua College of Profession and Technology, Jinhua, Zhejiang 321017, People’s Republic of China

## Abstract

The hydro­thermal reaction of CoCO_3_ and 4-fluoro­benzoic acid afforded the title Co^II^ complex, [Co(C_7_H_4_FO_2_)_2_(H_2_O)_2_]_*n*_. The Co^II^ atom is located on an inversion center and is coordinated by six O atoms from two water mol­ecules and four μ_2_-carboxyl­ate groups of 4-fluoro­benzoate anions, forming a distorted CoO_6_ octa­hedron, with Co—O bond lengths in the range 2.071 (2)–2.130 (2) Å. All adjacent O—Co—O angles are in the range 84.78 (6)–95.22 (6)° and opposite angles are 180.0°. Each μ-carboxyl­ate group of the 4-fluoro­benzoate anions bridges two symmetry-related Co^II^ atoms. Hydrogen-bonding inter­actions of the coordinated water mol­ecules further connect the cobalt–carboxyl­ate units, forming layers perpendicular to the *a* axis. The cobalt–oxygen layers are encased in a sandwich-like fashion by layers of π-stacked 4-fluoro­benzoate anions. Within these layers the benzene rings of the 4-fluoro­benzoate anions are π-stacked, with centroid–centroid distances of 3.432 (4) Å.

## Related literature

For other complexes of the 2(or 4)-fluoro­benzoato ligand, see: Zhang (2006 ▶ *c*); Zhang *et al.* (2005*a* ▶,*b* ▶). For related structures, see: Zhang (2004 ▶, 2005 ▶, 2006*a* ▶,*b* ▶,*c* ▶); Zhang *et al.* (2008 ▶); Majumder *et al.* (2006 ▶); Shi *et al.* (1996 ▶).
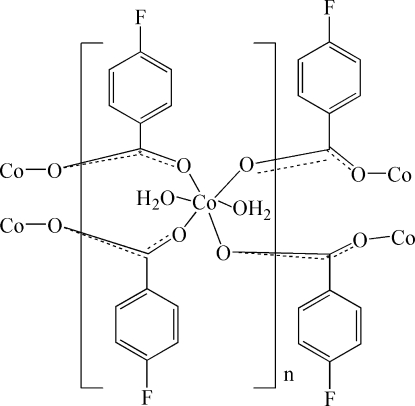


## Experimental

### 

#### Crystal data

[Co(C_7_H_4_FO_2_)_2_(H_2_O)_2_]
*M* *_r_* = 373.17
Monoclinic, 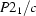

*a* = 14.866 (3) Å
*b* = 6.6043 (13) Å
*c* = 7.3081 (15) Å
β = 100.94 (3)°
*V* = 704.5 (3) Å^3^
*Z* = 2
Mo *K*α radiation
μ = 1.27 mm^−1^
*T* = 290 K
0.54 × 0.35 × 0.10 mm

#### Data collection

Rigaku R-AXIS RAPID diffractometer
Absorption correction: multi-scan (*ABSCOR*; Higashi, 1995 ▶) *T*_min_ = 0.590, *T*_max_ = 0.879
6437 measured reflections
1616 independent reflections
1432 reflections with *I* > 2σ(*I*)
*R*_int_ = 0.038

#### Refinement

*R*[*F* ^2^ > 2σ(*F* ^2^)] = 0.032
*wR*(*F* ^2^) = 0.087
*S* = 1.15
1616 reflections
106 parameters
H-atom parameters constrained
Δρ_max_ = 0.44 e Å^−3^
Δρ_min_ = −0.34 e Å^−3^

### 

Data collection: *RAPID-AUTO* (Rigaku, 1998 ▶); cell refinement: *RAPID-AUTO*; data reduction: *CrystalStructure* (Rigaku/MSC, 2002 ▶); program(s) used to solve structure: *SHELXS97* (Sheldrick, 2008 ▶); program(s) used to refine structure: *SHELXL97* (Sheldrick, 2008 ▶); molecular graphics: *SHELXTL* (Sheldrick, 2008 ▶); software used to prepare material for publication: *SHELXL97*.

## Supplementary Material

Crystal structure: contains datablocks I, global. DOI: 10.1107/S1600536809014913/zl2189sup1.cif

Structure factors: contains datablocks I. DOI: 10.1107/S1600536809014913/zl2189Isup2.hkl

Additional supplementary materials:  crystallographic information; 3D view; checkCIF report

## Figures and Tables

**Table 1:** Hydrogen-bond geometry (Å, °)

*D*—H⋯*A*	*D*—H	H⋯*A*	*D*⋯*A*	*D*—H⋯*A*
O2—H2*B*⋯O3^i^	0.85	2.00	2.833 (2)	167
O2—H2*A*⋯O3^ii^	0.85	2.11	2.835 (2)	143
O2—H2*A*⋯O1^iii^	0.85	2.42	3.115 (1)	140

Symmetry codes: (i) 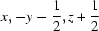; (ii) 
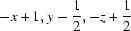
; (iii) 
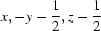
.

**Fig. 1.**
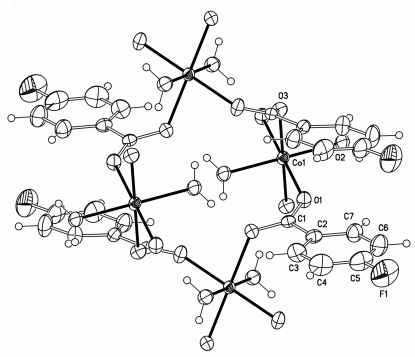
The structure unit of the title compound, showing the atom-labelling scheme. Displacement ellipsoids are drawn at the 50% probability level.

**Fig. 2.**
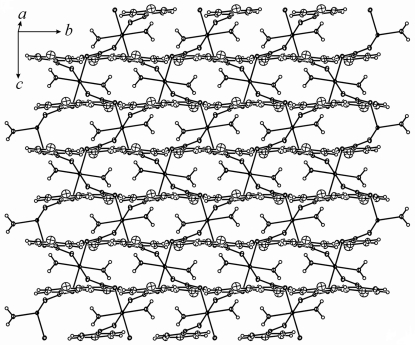
View of the title complex along the [100] direction showing the two-dimensional layering.

**Fig. 3.**
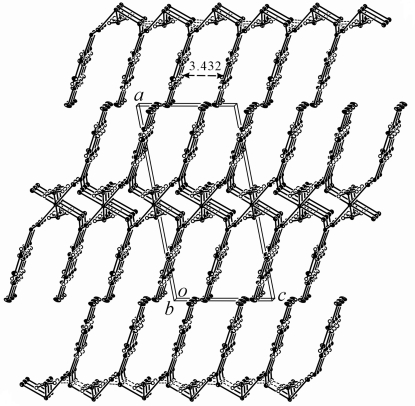
A packing diagram of the title complex, viewed along the b axis. π-π Stacking interactions are indicated as dashed double arrows.

## supplementary crystallographic information

### Comment

Cobalt(II) ions can form, among others, mononuclear and one-dimensional network complexes (Majumder *et al.*,2006). In this context we have studied and reported the crystal structures of complexes with halobenzoate ligands, X—C_6_H_4_COO^-^, where X is F, Cl, Br or I, (Zhang, 2004, 2005, 2006*a*,b,c; Zhang *et al.*, 2005, 2008). In this report we would like to report the synthesis and crystal structure of the title complex, ^2^∞[Co(H_2_O)_2_(FC_6_H_4_COO)_4/2_]. Within the title compound, each Co^II^ atom is located on a crystallographic inversion center and is coordinated by six O atoms from two water molecules and four µ_2_-carboxyl groups of 4-fluorobenzoic acid anions, to form a distorted CoO_6_ octahedron, with Co—O bond lengths in the range of 2.071 (2) to 2.130 (2) Å. All adjacent O—Co—O bond angles are in the range of 84.78 (6)–95.22 (6)° and opposite angles are 180.0 (1)°.

Each µ_2_-carboxyl group of the 4-fluorobenzoic anions bridges two symmetry related cobalt atoms, Co(1) and Co(1)^vi^ (vi: -*x* + 1, -*y*, -*z* + 1). Hydrogen bonding interactions of the coordinated water molecules further connect the cobalt-carboxylate units with each other to form layers perpendicular to the *a* axis (Fig.2). The O—H···O bond lengths are in the range of 2.83 (2) to 3.12 (1) Å, the O—H···O bond angles are in the range of 139.7 (1) —167.0 (1)°, Table 2. The cobalt-oxygen layers are encased in a sandwich like fashion by layers of π-stacked 4-fluorobenzoate anions. Within these layers τhe the benzene rings of the 4-fluorobenzoate anions are π stacked with centroid to centroid^iii^ (iii = x, 0.5-y, -0.5+z) distances of 3.432 (4)Å.

### Experimental

CoCO_3_ (0.132 g, 1.110 mmol), 4-fluorobenzoic acid (0.085 g, 0.607 mmol) and 15 ml CH_3_OH/H_2_O (1:2, *v*/*v*) were mixed and stirred for *ca* 5.0 h, and the resulting suspension was heated in a 23 ml Teflon-lined stainless steel autoclave at 433 K for 6 days. After the autoclave cooled to room temperature, the solid was filtered off. The resulting purple filtrate was allowed to stand at room temperature and slow evaporation over three months gave red block crystals suitable for X-ray analysis. Yield: 76%.

### Refinement

C-bound H atoms were placed in calculated positions, with C—H = 0.93Å and *U*_iso_(H) = 1.2*U*_eq_(C), and were refined using the riding-model approximation. The H atoms of the water molecule were located in a difference Fourier map and refined with an O—H distance restraint of 0.85 (1) Å and *U*_iso_(H) = 1.5*U*_eq_(O).

### Crystal data

**Table:**

[Co(C_7_H_4_FO_2_)_2_(H_2_O)_2_]	*F*(000) = 378
*M**_r_* = 373.17	*D*_x_ = 1.759 Mg m^−^^3^
Monoclinic, *P*2_1_/*c*	Mo *K*α radiation, λ = 0.71073 Å
Hall symbol: -P 2ybc	Cell parameters from 5552 reflections
*a* = 14.866 (3) Å	θ = 3.4–27.5°
*b* = 6.6043 (13) Å	µ = 1.27 mm^−^^1^
*c* = 7.3081 (15) Å	*T* = 290 K
β = 100.94 (3)°	Block, red
*V* = 704.5 (3) Å^3^	0.54 × 0.35 × 0.10 mm
*Z* = 2	

### Data collection

**Table:**

Rigaku R-AXIS RAPID diffractometer	1616 independent reflections
Radiation source: fine-focus sealed tube	1432 reflections with *I* > 2σ(*I*)
graphite	*R*_int_ = 0.038
Detector resolution: 10 pixels mm^-1^	θ_max_ = 27.5°, θ_min_ = 3.4°
ω scans	*h* = −19→19
Absorption correction: multi-scan (*ABSCOR*; Higashi, 1995)	*k* = −7→8
*T*_min_ = 0.590, *T*_max_ = 0.879	*l* = −9→9
6437 measured reflections	

### Refinement

**Table:**

Refinement on *F*^2^	Primary atom site location: structure-invariant direct methods
Least-squares matrix: full	Secondary atom site location: difference Fourier map
*R*[*F*^2^ > 2σ(*F*^2^)] = 0.032	Hydrogen site location: inferred from neighbouring sites
*wR*(*F*^2^) = 0.087	H-atom parameters constrained
*S* = 1.15	*w* = 1/[σ^2^(*F*_o_^2^) + (0.0337*P*)^2^ + 0.6464*P*] where *P* = (*F*_o_^2^ + 2*F*_c_^2^)/3
1616 reflections	(Δ/σ)_max_ < 0.001
106 parameters	Δρ_max_ = 0.44 e Å^−^^3^
0 restraints	Δρ_min_ = −0.34 e Å^−^^3^

### Special details

**Table:**

Geometry. All e.s.d.'s (except the e.s.d. in the dihedral angle between two l.s. planes) are estimated using the full covariance matrix. The cell e.s.d.'s are taken into account individually in the estimation of e.s.d.'s in distances, angles and torsion angles; correlations between e.s.d.'s in cell parameters are only used when they are defined by crystal symmetry. An approximate (isotropic) treatment of cell e.s.d.'s is used for estimating e.s.d.'s involving l.s. planes.
Refinement. Refinement of *F*^2^ against ALL reflections. The weighted *R*-factor *wR* and goodness of fit *S* are based on *F*^2^, conventional *R*-factors *R* are based on *F*, with *F* set to zero for negative *F*^2^. The threshold expression of *F*^2^ > σ(*F*^2^) is used only for calculating *R*-factors(gt) *etc*. and is not relevant to the choice of reflections for refinement. *R*-factors based on *F*^2^ are statistically about twice as large as those based on *F*, and *R*- factors based on ALL data will be even larger.

### Fractional atomic coordinates and isotropic or equivalent isotropic displacement parameters (Å^2^)

**Table:**

	*x*	*y*	*z*	*U*_iso_*/*U*_eq_	
Co1	0.5000	0.0000	0.5000	0.01947 (14)	
O1	0.60194 (11)	0.0944 (2)	0.7171 (2)	0.0278 (3)	
O2	0.56707 (11)	−0.2799 (2)	0.5136 (2)	0.0285 (3)	
H2B	0.5729	−0.3586	0.6071	0.043*	
H2A	0.5482	−0.3444	0.4130	0.043*	
O3	0.57083 (10)	0.0875 (2)	0.2851 (2)	0.0260 (3)	
F1	0.98672 (12)	0.3394 (4)	1.1685 (3)	0.0776 (7)	
C1	0.62472 (14)	0.2606 (3)	0.7934 (3)	0.0202 (4)	
C2	0.72091 (14)	0.2833 (4)	0.8984 (3)	0.0255 (4)	
C3	0.75685 (19)	0.4727 (4)	0.9486 (4)	0.0378 (6)	
H3	0.7203	0.5873	0.9217	0.045*	
C4	0.8473 (2)	0.4922 (5)	1.0391 (5)	0.0504 (8)	
H4	0.8725	0.6190	1.0720	0.060*	
C5	0.89849 (18)	0.3206 (5)	1.0786 (4)	0.0487 (7)	
C6	0.86542 (18)	0.1311 (5)	1.0332 (4)	0.0476 (7)	
H6	0.9021	0.0173	1.0633	0.057*	
C7	0.77507 (17)	0.1134 (4)	0.9404 (4)	0.0358 (5)	
H7	0.7509	−0.0139	0.9063	0.043*	

### Atomic displacement parameters (Å^2^)

**Table:**

	*U*^11^	*U*^22^	*U*^33^	*U*^12^	*U*^13^	*U*^23^
Co1	0.0219 (2)	0.0161 (2)	0.0191 (2)	−0.00099 (14)	0.00055 (14)	0.00070 (13)
O1	0.0262 (8)	0.0231 (8)	0.0306 (8)	−0.0007 (6)	−0.0034 (6)	−0.0042 (6)
O2	0.0390 (9)	0.0198 (8)	0.0254 (8)	0.0022 (6)	0.0027 (6)	0.0007 (6)
O3	0.0287 (8)	0.0240 (8)	0.0249 (7)	−0.0049 (6)	0.0041 (6)	0.0012 (6)
F1	0.0302 (9)	0.1146 (19)	0.0775 (14)	−0.0175 (11)	−0.0163 (9)	−0.0037 (13)
C1	0.0217 (9)	0.0223 (10)	0.0169 (9)	−0.0031 (8)	0.0045 (7)	0.0005 (7)
C2	0.0237 (10)	0.0312 (12)	0.0212 (10)	−0.0044 (9)	0.0026 (8)	−0.0023 (8)
C3	0.0354 (13)	0.0341 (14)	0.0425 (15)	−0.0079 (10)	0.0036 (11)	−0.0060 (11)
C4	0.0389 (15)	0.055 (2)	0.0544 (18)	−0.0201 (14)	0.0005 (13)	−0.0115 (13)
C5	0.0241 (12)	0.079 (2)	0.0398 (15)	−0.0120 (13)	−0.0031 (10)	−0.0034 (14)
C6	0.0291 (13)	0.0597 (19)	0.0491 (16)	0.0079 (12)	−0.0049 (11)	0.0031 (14)
C7	0.0300 (12)	0.0362 (14)	0.0378 (13)	0.0008 (10)	−0.0024 (9)	−0.0020 (10)

### Geometric parameters (Å, °)

**Table:**

Co1—O1^i^	2.0712 (16)	C1—C2	1.497 (3)
Co1—O1	2.0712 (16)	C2—C7	1.381 (3)
Co1—O2^i^	2.0938 (16)	C2—C3	1.382 (3)
Co1—O2	2.0938 (16)	C3—C4	1.387 (4)
Co1—O3	2.1301 (15)	C3—H3	0.9300
Co1—O3^i^	2.1301 (15)	C4—C5	1.365 (5)
O1—C1	1.248 (3)	C4—H4	0.9300
O2—H2B	0.8500	C5—C6	1.362 (5)
O2—H2A	0.8500	C6—C7	1.390 (4)
O3—C1^ii^	1.278 (3)	C6—H6	0.9300
F1—C5	1.357 (3)	C7—H7	0.9300
C1—O3^iii^	1.278 (3)		
			
O1^i^—Co1—O1	180.00 (10)	O1—C1—C2	117.95 (19)
O1^i^—Co1—O2^i^	87.54 (6)	O3^iii^—C1—C2	118.37 (18)
O1—Co1—O2^i^	92.46 (6)	C7—C2—C3	119.8 (2)
O1^i^—Co1—O2	92.46 (6)	C7—C2—C1	119.5 (2)
O1—Co1—O2	87.54 (6)	C3—C2—C1	120.7 (2)
O2^i^—Co1—O2	180.00 (9)	C2—C3—C4	120.1 (3)
O1^i^—Co1—O3	84.78 (6)	C2—C3—H3	120.0
O1—Co1—O3	95.22 (6)	C4—C3—H3	120.0
O2^i^—Co1—O3	91.33 (6)	C5—C4—C3	118.4 (3)
O2—Co1—O3	88.67 (6)	C5—C4—H4	120.8
O1^i^—Co1—O3^i^	95.22 (6)	C3—C4—H4	120.8
O1—Co1—O3^i^	84.78 (6)	F1—C5—C6	118.3 (3)
O2^i^—Co1—O3^i^	88.67 (6)	F1—C5—C4	118.4 (3)
O2—Co1—O3^i^	91.33 (6)	C6—C5—C4	123.4 (2)
O3—Co1—O3^i^	180.00 (6)	C5—C6—C7	117.8 (3)
C1—O1—Co1	134.73 (14)	C5—C6—H6	121.1
Co1—O2—H2B	123.6	C7—C6—H6	121.1
Co1—O2—H2A	109.0	C2—C7—C6	120.6 (3)
H2B—O2—H2A	110.8	C2—C7—H7	119.7
C1^ii^—O3—Co1	124.82 (13)	C6—C7—H7	119.7
O1—C1—O3^iii^	123.68 (19)		

Symmetry codes: (i) −*x*+1, −*y*, −*z*+1; (ii) *x*, −*y*+1/2, *z*−1/2; (iii) *x*, −*y*+1/2, *z*+1/2.

### Hydrogen-bond geometry (Å, °)

**Table:**

*D*—H···*A*	*D*—H	H···*A*	*D*···*A*	*D*—H···*A*
O2—H2B···O3^iv^	0.85	2.00	2.833 (2)	167
O2—H2A···O3^v^	0.85	2.11	2.835 (2)	143
O2—H2A···O1^vi^	0.85	2.42	3.115 (1)	140

Symmetry codes: (iv) *x*, −*y*−1/2, *z*+1/2; (v) −*x*+1, *y*−1/2, −*z*+1/2; (vi) *x*, −*y*−1/2, *z*−1/2.
